# Transgenic livestock for agriculture and biomedical applications

**DOI:** 10.1186/1753-6561-8-S4-O29

**Published:** 2014-10-01

**Authors:** Charles Long

**Affiliations:** 1 Texas A&M University, College Station, TX, USA

## 

The classical definition of a genetically engineered or "transgenic" animal would be "those animals modified by recombinant DNA (rDNA) techniques" according to the U.S. Food and Drug Administration (United States Food and Drug Administration, Guidance #187, 2009). Thus, the introduction or deletion of any gene by using recombinant DNA would fall into this category. Since the first reports of stable germline transmission of integrated DNA in 1982, hundreds of thousands of transgenic animals have been produced, the vast majority of which are mice used in biomedical research. However, there are also thousands of agrarian and aquaculture species that have been produced for a wide variety of purposes from production of human biopharmaceuticals, xenotransplantation and to enhance economically important production traits.

We can separate the transgenic animals produced in our laboratory into 3 major classes; production agriculture, biopharmaceutical production in milk and biomedical models of human disease. This brief review will highlight some of those projects in each category.

## Transgenic livestock in production agriculture

Production agriculture projects in our laboratory have utilized RNA interference to enhance meat production through improved muscle growth. Myostatin is a negative regulator of muscle cell proliferation during fetal development. It is know that inactivating mutations in a number of species, including cattle, develop a muscle overgrowth phenotype [1]. Although the increased muscle can be considered a positive trait, the increase in dystocia related to the size of the calves at birth limit the utilization of these naturally occurring myostatin mutations in production agriculture. RNA interference has been utilized to overexpress a short hairpin RNA (shRNA) with homology to the myostatin mRNA *in vivo*. Direct injection into the perivitelline space of bovine zygotes with recombinant lentiviral vectors promoted the efficient integration of the shRNA expression constructs into the bovine genome. The expression and cellular processing of the myostatin shRNA led to degradation of the myostatin mRNA and an overt muscling phenotype in transgenic cattle [2]. Subsequent studies in pigs also show a significant increase in fetal pig weights compared to transgenic controls. The advantage of this approach is that shRNA mediated degradation of the myostatin mRNA over a range of values, typically between 20-80% of controls. This approach can be used to decrease the production of myostatin in a way that may lead to increase muscling without associated dystocia problems.

Enhanced resistance to endemic diseases, especially where vaccination strategies are ineffective or difficult to achieve, could dramatically increase the production capacity and thus the profitability of animal production systems. We have used RNAi to investigate host/pathogen interactions by direct suppression of viral proteins or immune system modulators, which decrease viral replication and improve disease resistance. Direct targeting of viral mRNA can reduce but not eliminate vesicular stomatitis virus or equine infectious anemia virus *in vitro *[3]. Likewise, suppression of the immunomodulatory components of the innate immune system have shown positive results, but not complete protection of the cells from viral infection. Current and future work will continue to refine these efforts with the long term goal of producing animals with enhanced innate immunity to a wide variety of viral and bacterial pathogens. Short-term investigations in collaboration with the USDA Plum Island Animal Disease Center have focused on therapeutic approaches to prevent spread of diseases such as Foot and Mouth Disease Virus during outbreaks.

## Transgenic livestock for biopharmaceutical production

Mammals have the remarkable ability to produce large quantities of proteins in their mammary glands and secrete these proteins during lactation. This natural protein production "factory" can be utilized to produce proteins that benefit both animals and humans through genetic engineering. By placing a mammary gland specific promoter in a position to express a protein of interest, the mammary gland can be made to produce nearly any protein desired [4]. Production of useful biopharmaceutical proteins in the milk of several species has been demonstrated. Species such as mice, rabbits, pigs, goats and cattle have all been utilized to produce proteins of biomedical value in milk. This is a low cost, high yield system that can be implemented to produce biopharmaceuticals without the hundreds of millions of dollars necessary to build cell culture or bacterial processing facilities. In partnership with rEVO Biologics our laboratory has reinitiated a project to produce a malaria vaccine antigen in the milk of goats.

In collaboration with rEVO Biologics, Texas A&M has rederived a line of goats, originally produced through pronuclear microinjection, that produce a tagged malaria parasite protein exclusively in their milk. At the beginning of this project, scientists at rEVO inserted a modified portion of a malaria parasite protein (MSP1_42_) into mice and collected the milk. MSP1_42 _was purified and mixed with an adjuvant to create an immune response that could mitigate a malaria exposure/challenge [5]. This vaccine was tested in non-human primates and effectively protected the animals from a lethal dose of malaria parasite. Thus, it was clear that the MSP1_42 _was capable of producing effective vaccine. To increase production, rEVO inserted rDNA encoding the same protein into the genome of a dairy goat and produced animals capable of making the exact same protein on a much larger scale. In fact, one goat during her lactation can produce enough MSP1_42 _to produce approximately 1 million doses of malaria vaccine. These goats can now be propagated through natural or artificial insemination to expand the herd. Ongoing research will establish whether the goat derived material has the same biological activity as the previously tested mouse derived antigen. If so, this represents a low cost method of producing high quality antigen which can be readily purified to produce vaccine.

## Transgenic livestock as biomedical models of human disease

In addition to the benefits livestock animals bring to the human population in terms of food and fiber, they also represent interesting models to study diseases important to both human and veterinary medicine. Although naturally occurring genetic mutations have been discovered that produce important models of human disease, the ability to specifically modulate gene expression in livestock represents a unique opportunity to study gene function in animals. Livestock, especially pigs, share a similar size and physiology to humans and are important subjects for study of potential disease therapies prior to undergoing costly clinical trials. Our lab has begun to develop pigs as biomedical models for human obesity and metabolic syndrome, by inducible tissue specific expression of transgenes delivered by recombinant retroviral vectors.

Retroviral vectors were utilized to produce the first transgenic mice. Although these initial experiments were successful, problems with germline transmission hindered further utilization for many years. Since those initial studies, a multitude of innovations have produced a new class of recombinant, replication incompetent lentiviruses that have overcome many of the initial limitations. These highly modified and updated vectors are rapidly being utilized in human gene therapy trials with good results. Thus, lentiviral vectors are recognized as a safe and effective means for transduction of a number of cell types including gametes and embryos. Microinjection of concentrated lentiviral particles into the perivitelline space of livestock oocytes or zygotes can produce genetically modified embryos at high rates [2,6]. In the context of transgenic livestock, lentiviral mediated delivery of transgenes represents a unique regulatory obstacle due the nature of the integrated construct. Thus, in the short term, utility as a vector for agricultural applications would appear limited at best. Nonetheless, many biomedical applications can utilize the ability of these vectors to package and deliver transgenes into oocytes or zygotes to efficiently produce genetically modified embryos for subsequent transfer and production of offspring.

Our laboratory is currently developing new recombinant lentiviruses capable of tetracycline inducible, tissue specific expression of transgenes in livestock species. In contrast to the murine model, there are currently limited options for this type of transgene expression systems in swine or other livestock species. These constructs can be designed to overexpress proteins, suppress endogenous translation via RNAi or both. The goal of the current work is to target stearyl Co-A desaturase (SCD-1), an enzyme that is a key regulatory element in lipid biosynthesis [7]. By altering expression of SCD-1 in a tissue and inducible way, we can control tissue steatosis. Muscle and hepatic steatosis is detrimental to human health, but is considered a valuable production trait in cattle muscle (marbling). Thus, the animals produced in these studies will have utility as biomedical models, but also could be useful in livestock production systems. The further refinement of inducible, tissue specific methods of genetic engineering offer exceptional opportunities for production of biomedical models of human disease and livestock production.

## Summary

As new genetic engineering technologies develop, the ability to effectively and efficiently engineer the genome of livestock species will improve. Recently the utilization of site specific nucleases (TALEN and ZFN) and RNA guided nucleases (CRISPR/Cas9) have revolutionized the field of genetic engineering [8,9]. Application of these techniques are underway in our laboratory as well as many others in order to impart unique traits to livestock. These traits will lead to improved production of food and fiber as well as produce animals capable of making biopharmaceuticals and nutriceuticals. We are reaching the point in livestock genetic engineering where the technology is no longer the limiting factor. We are now only limited by our imaginations and the burdensome regulatory pathways for bringing these products to the world's population.
